# Cognitive Behavioral Therapy for Symptom Preoccupation Among Patients With Premature Ventricular Contractions: Nonrandomized Pretest-Posttest Study

**DOI:** 10.2196/53815

**Published:** 2024-05-07

**Authors:** Björn E Liliequist, Josefin Särnholm, Helga Skúladóttir, Eva Ólafsdóttir, Brjánn Ljótsson, Frieder Braunschweig

**Affiliations:** 1 Department of Clinical Neuroscience Division of Psychology Karolinska Institutet Stockholm Sweden; 2 Department of Medicine Cardiology Unit Karolinska Institutet Stockholm Sweden; 3 Department of Cardiology Karolinska University Hospital Stockholm Sweden

**Keywords:** premature ventricular contractions, quality of life, symptom preoccupation, cognitive behavioral therapy: CBT

## Abstract

**Background:**

Premature ventricular contractions (PVCs) are a common cardiac condition often associated with disabling symptoms and impaired quality of life (QoL). Current treatment strategies have limited effectiveness in reducing symptoms and restoring QoL for patients with PVCs. Symptom preoccupation, involving cardiac-related fear, hypervigilance, and avoidance behavior, is associated with disability in other cardiac conditions and can be effectively targeted by cognitive behavioral therapy (CBT).

**Objective:**

The aim of this study was to evaluate the effect of a PVC-specific CBT protocol targeting symptom preoccupation in patients with symptomatic idiopathic PVCs.

**Methods:**

Nineteen patients diagnosed with symptomatic idiopathic PVCs and symptom preoccupation underwent PVC-specific CBT over 10 weeks. The treatment was delivered by a licensed psychologist via videoconference in conjunction with online text-based information and homework assignments. The main components of the treatment were exposure to cardiac-related symptoms and reducing cardiac-related avoidance and control behavior. Self-rated measures were collected at baseline, post treatment, and at 3- and 6-month follow-ups. The primary outcome was PVC-specific QoL at posttreatment assessment measured with a PVC-adapted version of the Atrial Fibrillation Effects on Quality of Life questionnaire. Secondary measures included symptom preoccupation measured with the Cardiac Anxiety Questionnaire. PVC burden was evaluated with 5-day continuous electrocardiogram recordings at baseline, post treatment, and 6-month follow-up.

**Results:**

We observed large improvements in PVC-specific QoL (Cohen *d*=1.62, *P<.*001) and symptom preoccupation (Cohen *d*=1.73, *P*<.001) post treatment. These results were sustained at the 3- and 6-month follow-ups. PVC burden, as measured with 5-day continuous electrocardiogram, remained unchanged throughout follow-up. However, self-reported PVC symptoms were significantly lower at posttreatment assessment and at both the 3- and 6-month follow-ups. Reduction in symptom preoccupation had a statistically significant mediating effect of the intervention on PVC-specific QoL in an explorative mediation analysis.

**Conclusions:**

This uncontrolled pilot study shows preliminary promising results for PVC-specific CBT as a potentially effective treatment approach for patients with symptomatic idiopathic PVCs and symptom preoccupation. The substantial improvements in PVC-specific QoL and symptom preoccupation, along with the decreased self-reported PVC-related symptoms warrant further investigation in a larger randomized controlled trial.

**Trial Registration:**

ClinicalTrials.gov NCT05087238; https://clinicaltrials.gov/study/NCT05087238

## Introduction

Premature ventricular contractions (PVCs) are a common type of cardiac arrhythmia with a prevalence of 69%-99.5% in the adult population [1,2]. PVCs are commonly asymptomatic but can result in mild to disabling symptoms due to palpitations, dyspnea, presyncope, and fatigue [3]. In the absence of structural heart disease or inherited ion channelopathies, PVCs are referred to as idiopathic and considered benign, although a high PVC burden may induce a cardiomyopathy in some patients [3,4]. Medical treatment with beta-blockers or nondihydropyridine calcium channel blockers can decrease the PVC burden and provide symptomatic improvement; however, this treatment is ineffective in a large portion of patients and may cause side effects. Catheter ablation is the most effective approach to abolish PVCs and has been emphasized in recent guidelines as the recommended first-line treatment for symptomatic idiopathic PVCs [5]. However, ablation may not be readily available or may be offered with some reservation owing to the risk of rare but potentially life-threatening complications and considerable costs [5]. Therefore, many patients with PVCs continue to live with persistent and debilitating symptoms.

Symptom preoccupation, which involves excessive attention to symptoms, fear of symptoms, and associated avoidance behavior, is related to increased symptom severity and low disease-specific quality of life (QoL) in other somatic conditions [6,7]. Studies of atrial fibrillation (AF) have shown that symptom preoccupation, rather than the objective arrhythmia burden, explains elevated symptom severity and impaired QoL [8,9]. However, few studies have investigated QoL in patients with PVCs [10], and the factors underpinning symptom severity and QoL in these patients are not well understood. Given the similar symptom presentation as that associated with AF, we hypothesized that symptom preoccupation is likely to play a role in the subjective symptom experience for patients with PVCs. We have defined symptom preoccupation in the context of cardiac arrhythmias (AF and PVCs) as the fear of experiencing and triggering cardiac-related symptoms, hypervigilance toward cardiac symptoms, persistent worry about complications, and arrhythmia-related avoidance of physical and social activities [11,12]

Cognitive behavioral therapy (CBT) has been found to be effective in reducing disability associated with both anxiety disorders [13] and somatic conditions where symptom preoccupation is prevalent [6,7,13]. In a series of clinical studies, Särnholm et al [11,12,14] developed AF-specific CBT (AF-CBT) targeting symptom preoccupation, which resulted in significant improvements in AF-specific QoL and self-reported symptom severity. Therefore, the purpose of this study was to further adapt and evaluate the AF-CBT protocol to target symptom preoccupation in patients with symptomatic idiopathic PVCs.

PVC-specific CBT (PVC-CBT) aims to break the cycle of cardiac-related fear and hypervigilance as well as arrhythmia-specific avoidance behavior and disability through repeated exposure to cardiac-related symptoms and situations that have been avoided due to fear, negative emotional responses, and apprehension of experiencing PVCs. We also aimed to investigate changes in the objective arrythmia burden as measured with electrocardiogram (ECG), along with the role of symptom preoccupation as a potential mediator of the treatment effect on PVC-specific QoL.

## Methods

### Study Design

In this uncontrolled pilot trial, we used a pretest-posttest design with 3- and 6-month follow-ups. Self-rated measures were completed online using a secure web-based assessment tool and were collected at pretreatment, post treatment, and at 3- and 6-month follow-ups. The primary outcome and potential mediators were also collected weekly during treatment. The aim was to include 30 participants, yielding a power of 80% to detect at least a moderate increase in the main outcome measure, corresponding to an effect size of Cohen *d*=0.65. However, due to time constraints, we made a pragmatic decision to stop recruitment at 19 participants. This decision was based on the clinical impression of substantial improvements in the first 15 treated participants and experiences from the previous pilot study of AF-CBT, where large average improvement was observed in 19 participants [11].

### Participants

Participants were recruited nationwide from Sweden by advertisement in social media and newspapers. To be eligible for the study, participants had to fulfill the following inclusion criteria: (1) 18-70 years old, (2) diagnosed with PVCs with impairing or bothering PVC-associated cardiac symptoms, (3) on medication in accordance with current guidelines [5], and (4) able to read and write in Swedish. Participants were excluded if they had (1) any structural heart disease, including previous myocardial infarction, heart failure with preserved or reduced left ventricular ejection fraction, valvular disease, or previous cardiac surgery; (2) other arrhythmia or severe medical illness; (3) were scheduled for ablation therapy or any other cardiovascular intervention; (4) any medical restriction to physical exercise; (5) severe psychiatric disorder, severe depression, or risk of suicide; or (6) alcohol dependency. All participants underwent cardiac and psychological assessments to ensure that eligibility criteria were met. Participants were asked not to engage in other psychological treatment and only make necessary changes in medication during study participation. Participants were recruited and treated between January 2021 and October 2021, and the last 6-month follow-up was conducted in April 2022.

### Ethical Considerations

The Regional Ethics Review Board in Gothenburg approved the trial protocol (Dnr 2020–05809) and the study was registered on ClinicalTrials.gov (NCT05087238). The study was conducted in accordance with the Declaration of Helsinki and all respondents provided informed consent prior to their involvement in the study, wherein they were given detailed information regarding the study’s purpose, procedures, potential risks, benefits, and their rights as study participants. The informed consent form outlined these aspects clearly, and participants were given the opportunity to ask questions before agreeing to participate. All identifying data were stored on secure servers and data analysis was conducted on pseudonymized data. No compensation was given for participating. This study report adheres to the TREND (Transparent Reporting of Evaluations with Nonrandomized Designs) statement checklist for nonrandomized interventions [15]. The authors assure the completeness and accuracy of the data and adherence to the trial protocol.

### Procedure

Applicants registered at the study’s secure webpage and completed an online screening, including informed digital consent, demographic questions, and medical history. Applicants also completed the Alcohol Use Disorders Identification Test [16], the 9-item Patient Health Questionnaire (PHQ-9) measuring depressive symptoms [17], the Atrial Fibrillation Effects on Quality of Life (AFEQT) questionnaire adapted for PVCs (primary outcome measure), and the Cardiac Anxiety Questionnaire (CAQ) [18]. The cardiac study nurse (EÓ) then screened the applicants’ medical records and conducted clinical telephone interviews to ensure that eligibility criteria were met. Eligible patients then underwent a structured telephone-based psychological assessment by a clinical psychologist. All clinical assessments and the cardiac parameters from the medical chart were reviewed by the study cardiologist (HS) before a decision on inclusion was made.

### Intervention

The PVC-CBT intervention consisted of 10 weekly face-to-face sessions with a clinical psychologist (BEL) delivered via videoconference in conjunction with online text-based modules accessed through a secure web-based platform. All theoretical elements were presented verbally in sessions 1-4 and summarized in text-based form online together with homework assignments. This design allowed us to combine the flexibility of a face-to face session with the scaffolding structure of the internet-based format. The clinical psychologist could consult the study cardiologist in cases of uncertainties regarding participants’ health. Due to technical shortcomings of the videoconference application, some of the weekly sessions were delivered by telephone and one participant received all sessions by telephone. The last 6 sessions focused on continuing working with the central elements of treatment presented in sessions 1-4. Participants were recommended to spend 30 minutes a day on the homework assignments. The treatment was based on the AF-specific CBT protocol [11,12,14] and adapted to patients with PVCs by authors BEL and JS.

The PVC-CBT was designed to target symptom preoccupation (ie, fear and hypervigilance toward cardiac-related symptoms and avoidance behavior) and included the following interventions: (1) education on PVCs and common psychological reactions to PVC symptoms, and the role of control and avoidance behavior in maintaining fear and hypervigilance of PVC symptoms; (2) interoceptive exposure to physical sensations similar to PVC symptoms by performing physical exercises such as increasing the heart rate and inducing palpitations by running on the spot or inducing dyspnea by excessive breathing to reduce the fear of symptoms and hypervigilance; (3) self-observation of cardiac symptoms, thoughts, feelings, and behavioral impulses to reduce fear and hypervigilance, serving as a form of interoceptive exposure technique; (4) in vivo exposure to avoided activities that were anticipated to elicit or potentially exacerbate PVC symptoms (such as vigorous exercise) or situations in which PVC symptoms are unwanted (such as engaging in leisure activities or driving); and (5) strategies on how to refrain from behaviors that serve to control symptoms, such as pulse checking, and how to handle worry when conducting exposure exercises. Participants were encouraged to combine interventions 2-5 to maximize the effect of exposure (ie, inducing dyspnea by excessive breathing [interoceptive exposure] before taking a walk alone in the woods [in vivo exposure]) and then using self-observation while experiencing symptoms instead of checking their pulse. Participants were also encouraged to view symptomatic episodes as opportunities to practice the skills acquired in treatment. The last module focused on (6) relapse prevention, including participants making their own plan for continuous practice of the acquired skills after the end of treatment. See Textbox 1 for an overview of the treatment.

**Overview of the treatment plan in cognitive behavioral therapy for patients with premature ventricular contractions (PVCs).**

**Education**

- Education on PVCs
- The role of cardiac-related fear, hypervigilance, and behavior on symptoms and quality of life
- Self-observation of cardiac-related symptoms, thoughts, feelings, and behavioral impulses

**Interoceptive exposure**

- Exposure to physical sensations associated with PVC symptoms

**In vivo exposure**

- Gradual exposure to avoided situations or activities that patients fear may elicit or aggravate PVC symptoms or where symptoms are unwanted
- Combining in vivo exposure with interoceptive exposure while refraining from control and safety behavior

**Relapse prevention**

- Prevention of relapse into control or avoidance behavior by identifying risk situations

### Assessments

#### Design

All outcome measures were completed online, with no interference of study personnel, at pretreatment, post treatment, and at 3- and 6-month follow-ups, except when noted otherwise.

#### Primary Outcome

In the absence of a validated PVC-specific QoL measure, we used the AFEQT [19] as the primary outcome measure and adapted the questionnaire to PVCs (AFEQT-PVC). The AFEQT is a well-validated instrument for assessing self-reported AF-specific QoL in four domains: AF symptoms, impact on physical and social activities, medical treatment concerns, and satisfaction with AF treatment. The scale consists of 20 items with a total score ranging from 0 (severe AF symptoms and disability) to 100 (no AF symptoms and disability) [19]. The AFEQT has been shown to be sensitive to clinical change, with a change of 18.9 points corresponding to a meaningful improvement as assessed by a physician [19,20]. The original structure of the AFEQT questionnaire was preserved, only removing the last 6 items measuring satisfaction and concern with current medical treatment as these issues were not targeted in the study treatment. All other items were retained except for changing the wording relevant to AF to be relevant to PVCs (eg, “On a scale of 1 to 7, over the past 4 weeks, as a result of your extra heart beats*, how* much did the feelings below bother you?”). The AFEQT-PVC measures PVC-related QoL in the following domains: arrhythmia symptoms and impairment in physical and social activities. The adapted scale consists of 16 items (0-5), with a total score ranging from 0 (severe symptoms and disability) to 100 (no symptoms or disability).

#### Secondary Outcomes

Secondary outcome measures included the Symptoms Checklist (SCL), which consists of two subscales measuring the frequency and severity of arrhythmia-specific symptoms [21], and the CAQ, which was used to measure symptom preoccupation and consists of three subscales: (1) cardiac-related fear, (2) attention to cardiac-related symptoms, and (3) cardiac-related avoidance [18]. QoL was measured with the 12-item Short-Form Health Survey (SF-12), which contains two subscales measuring physical health–related QoL (PCS-12) and mental health–related QoL (MCS-12) [22]. To assess fear toward bodily symptoms, the Body Sensations Questionnaire (BSQ) [23] was used. Stress reactivity was measured with the Perceived Stress Scale (PSS-4) [24] and physical activity was measured with the Godin-Shepard Leisure Time Physical Activity Questionnaire (GSLTPAQ) [25]. Depressive symptoms were measured with the PHQ-9 [17] and general anxiety was measured with the General Anxiety Disorder scale (GAD-7) [26]. Treatment credibility was measured with the Credibility/Expectancy Scale [27] in the second week of treatment.

Patient satisfaction with the treatment was assessed with the Client Satisfaction Questionnaire (CSQ) [28] post treatment. Potential adverse events from treatment were assessed at post treatment and at 3- and 6-month follow-ups. Participants were instructed to report and rate the short- and long-term discomfort caused by the adverse event from 0 (did not affect me at all) to 3 (affected me very negatively) [6].

#### ECG Measurements of Objective PVC Burden

To assess changes in the objective PVC burden (number of PVCs per day), participants wore a three-channel ambulatory ECG patch (ePatch [29]) continuously for 5 days at pretreatment, post treatment, and at the 6-month follow-up. All participants contributed with three recordings except one participant who missed the 6-month follow-up recording.

As a measure of the subjective experience of the PVC burden, participants were instructed to indicate symptoms by tapping a button on the patch recorder when experiencing symptoms of PVCs. The patch recorder was delivered via mail together with written instructions. The ECG data were analyzed by an experienced consultant using specialized software (Cardiologs; Cardiologs Technologies). The consultant was blinded to participant ID and assessment occasion.

### Statistical Analysis

Linear mixed modeling was performed in Stata/IC 16.0 to analyze the change in the estimated mean for assessments from pretreatment to post treatment and from pretreatment to 3-month and 6-month follow-up, respectively. Effect sizes of within-group changes (Cohen *d*) were calculated as the mean change between the two compared time points (pretreatment to post treatment, pretreatment to 3-month follow-up, and pretreatment to 6-month follow-up) divided by each measure’s standard deviation at baseline. The 95% CIs for effect sizes were calculated in R [30] using bootstrapping with 5000 samples. Data were analyzed in an intention-to-treat design, meaning that all participants were included in the analyses regardless of treatment completion status.

The change in objective PVC burden and in the self-reported PVC burden (ie, indicating PVC symptoms) was analyzed using the ECG measurements from pretreatment to post treatment and from pretreatment to 6-month follow-up in Stata/IC 16.0. Profile analysis with a Poisson generalized estimation equation model and log-link function was used for the incidence rate of objective PVCs and self-reported PVCs by tapping the patch recorder device.

### Mediation Analysis

The potential mediating effects of symptom preoccupation (CAQ) on the effect of the treatment on the primary outcome measure were analyzed in an exploratory mediation analysis using the weekly version of the AFEQT-PVC, only differing from the AFEQT-PVC in that participants were asked to recall a period of 1 week instead of 1 month. The mediation analyses were conducted based on the 10 weekly measurements collected during the treatment (ie, at the beginning of the first treatment week until the beginning of the tenth treatment week).

The three subscales (attention, avoidance, and fear) as well as the total score of the CAQ were included as indicators of symptom preoccupation. To control for nonspecific improvement, we used the weekly versions of PSS-4 (perceived stress) and GSLTPAQ (physical activity), which are not targeted in the CBT treatment, as competing mediators. The analyses were performed in line with the procedure described by Baron and Kenny [31] and further developed by Preacher and Hayes [32]. With the purpose of investigating to what extent the changes in outcome could be explained by changes in the mediators, we conducted both single mediator analyses, in which each mediator was tested separately, and multiple mediator analyses, in which we included all mediators to compete. This design allowed us to study the relative contribution of each mediator to the improvement on the outcome weekly AFEQT-PVC.

We hypothesized a gradual and linear improvement in outcome during treatment with an effect of treatment week on mediators and the outcome. Further, we expected an association between the mediators and outcome during treatment. Both the single and multiple mediation analyses were performed in three steps. First, the association between treatment week and the mediator(s) (ie, a-path) was estimated. Second, while controlling for treatment week, the association between the mediator(s) and weekly AFEQT-PVC (ie, b-path) over the course of the therapy was estimated. Third, the ab-product, which is the indirect or mediated effect (ie, the contribution of the change in the mediator on the effect of treatment week on the outcome), was calculated by multiplying the a- and b-path estimates for each mediator. In the second set of analyses, which included all mediators, the first step was conducted separately for each mediator as the dependent variable and the second step included all mediators as independent variables. To account for dependency between the weekly measurements, all analyses were based on linear mixed models, with random intercept 95% CIs for the indirect effects (ie, the ab*-*products) estimated using 5000 bootstrap replications of all analyses; the criterion for a statistically significant mediation effect was that the 95% CI did not contain zero [32].

## Results

### Sample

**Figure 1:**
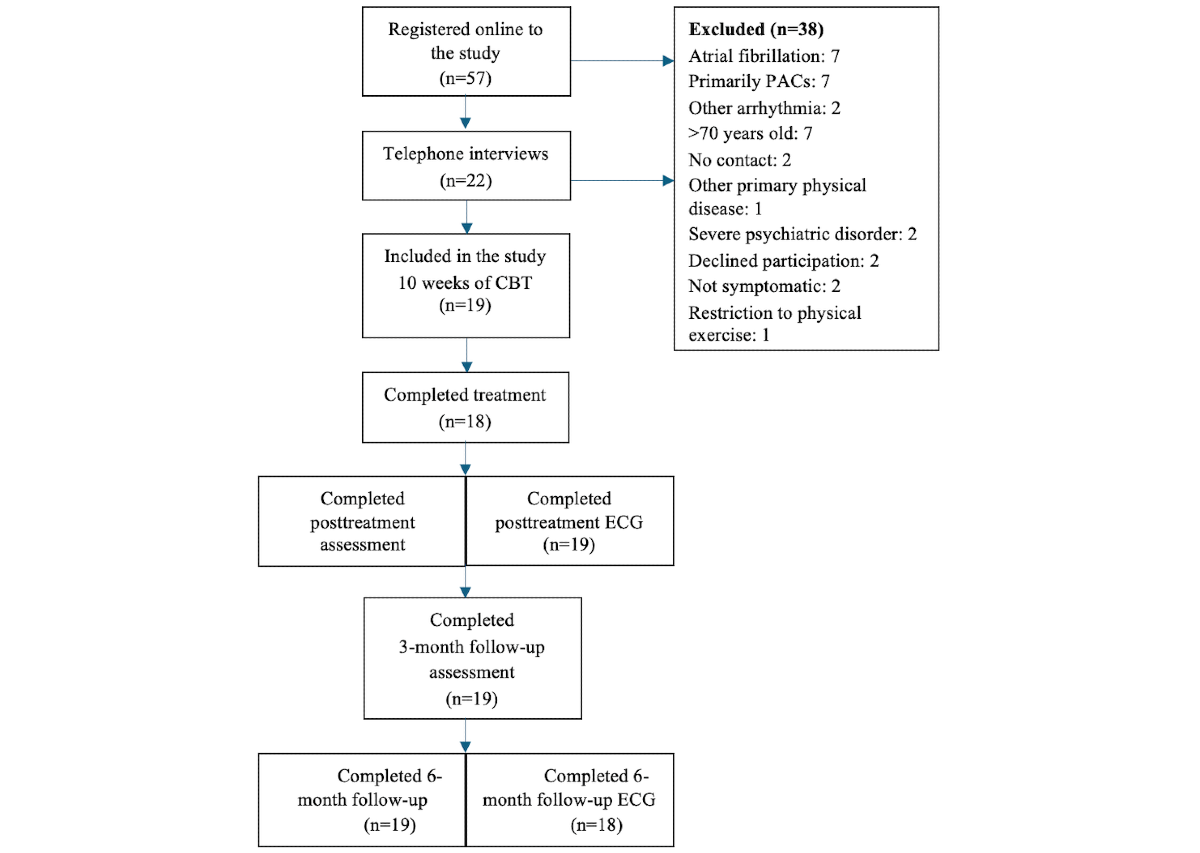
Flow of participants trough the trial. CBT: cognitive behavioral therapy; ECG: electrocardiogram; PAC: premature atrial contractions; PVC: premature ventricular contractions.

Table 1 displays the characteristics of the participants, and Figure 1 illustrates the participant flow through the trial. The included sample (N=19) predominantly comprised women (14/19, 74%). The mean age was 50.0 (SD 15.2) years and the self-reported mean time since the PVC diagnosis was 5 (SD 5.3) years.

**Table 1:** Characteristics of study participants at baseline (N=19).

Characteristics		Value
Women, n (%)		14 (74)
Age (years), mean (SD)		50 (15)
**Employment status, n (%)**		
	Employed	12 (63)
	Retired	5 (26)
	Self-employed	1 (5)
	Student	1 (5)
**Highest completed education, n (%)**		
	Secondary	4 (21)
	Tertiary	15 (79)
PVC^a^ duration (years), mean (SD)		5 (5)
**Current medication, n (%)**		
	Beta-blockers	11 (58)
	Calcium-channel blocker	2 (11)
	Antiarrhythmics	1 (5)
	ACEi^b^/ARB^c^	1 (5)
	Anticoagulation	1 (5)
	SSRI^d^	1 (5)
	Thyroid replacement therapy	3 (16)
**Medical disorders, n (%)**		
	Hypertension	2 (11)
	Dyslipidemia	2 (11)
	Obstructive sleep apnea	2 (11)
	Hypothyroidism	4 (21)
**Comorbid psychiatric conditions, n (%)**		
	Any psychiatric condition	13 (68)
	Depressed mood	2 (11)
	Excessive worry	4 (21)
	Social anxiety	3 (16)
	Panic attacks	2 (11)
	Agoraphobia	2 (11)
	Trauma-related stress symptoms	2 (11)
	Exhaustion symptoms	1 (5)
	Sleeping impairment	5 (26)
Previous psychological treatment, n (%)		13 (68)

^a^PVC: premature ventricular contraction.

^b^ACEi: angiotensin converter-enzyme inhibitor.

^c^ARB: angiotensin receptor blocker.

^d^SSRI: selective serotonin reuptake inhibitor.

### Treatment Activity

Mean session attendance was 9.6 (SD 1.9) sessions, ranging from 2 to 10 sessions. In total, 18 of the 19 participants (95%) were considered treatment completers, meaning that they completed at least 3 sessions and engaged in interoceptive and in vivo exposure exercises, and thus received the core components of the treatment. The one noncompleter attended 2 sessions. There were no missing self-assessment data at any assessment point, whereas one ECG measurement was missing at the 6-month follow-up.

### Primary and Secondary Outcomes

Table 2 shows the scores for the continuous outcomes at all assessment points. We observed substantial improvements in PVC-specific QoL (AFEQT-PVC), with large within-group effect sizes at the pretreatment-to-posttreatment assessment. Furthermore, we observed large pretreatment-to-posttreatment reductions in the self-reported frequency (SCL frequency) and severity (SCL severity) of arrhythmia symptoms, and in symptom preoccupation as measured by the total CAQ score as well as on all three subscales of the CAQ: fear, avoidance, and attention. Large effect sizes were also observed for mental health–related QoL (MCS-12), bodily symptoms (BSQ), and depressive symptoms (PHQ-9). Moderate effect sizes were observed on self-perceived stress (PSS-4), general anxiety (GAD-7), and physical activity (GSLTPAQ). One participant (1/19, 5%) reported substantially higher levels of physical activity than the others, leading to a 7-fold increase in variance on the GSLTPAQ. This participant was deemed an outlier and removed from the outcome analysis. No significant effect was seen on the physical health–related QoL (PCS-12). Results for all measures were sustained at the 3- and 6-month follow-ups compared with baseline assessments, except for the PSS-4 score that was nonsignificant at 3-month follow-up.

**Table 2:** Continuous treatment outcome measures and mixed-effects regression model results.

Measure and assessment time point			Mean (SD)		Change from pretreatment	
				Cohen *d*^a^ (95% CI)^b^		*P* value
**AFEQT-PVC^c^**						
	Pretest	58.3 (13.8)		—^d^		—
	Posttest	80.7 (16.1)		1.62 (1.07 to 2.28)		<.001
	3-month follow-up	81 (17.5)		1.64 (1.06 to 2.36)		<.001
	6-month follow-up	79.5 (19.4)		1.53 (0.92 to 2.18)		<.001
**SC^e^ frequency**						
	Pretest	19.6 (7.8)		—		—
	Posttest	13 (7.2)		0.84 (0.47 to 1.2)		<.001
	3-month follow-up	12.3 (7.1)		0.93 (0.51 to 1.33)		<.001
	6-month follow-up	12.2 (8.0)		0.94 (0.52 to 1.4)		<.001
**SCL severity**						
	Pretest	18.2 (6.4)		—		—
	Posttest	10 (6.1)		1.14 (0.75 to 1.61)		<.001
	3-month follow-up	10 (6.1)		1.29 (0.84 to 1.76)		<.001
	6-month follow-up	9.9 (5.8)		1.30 (0.89 to 1.81)		<.001
**CAQ^f^**						
	Pretest	35.6 (9.2)		—		—
	Posttest	19.6 (9.9)		1.73 (1.27 to 2.38)		<.001
	3-month follow-up	18.8 (0.5)		1.82 (1.26 to 2.39)		<.001
	6-month follow-up	19.7 (12.9)		1.71 (1.03 to 2.54)		<.001
**CAQ fear**						
	Pretest	16.5 (5.3)		—		—
	Posttest	9.9 (5.3)		1.22 (0.78 to 1.71)		<.001
	3-month follow-up	9.3 (5.3)		1.35 (0.94 to 1.87)		<.001
	6-month follow-up	10.3 (6.4)		1.16 (0.67 to 1.78)		<.001
**CAQ avoid**						
	Pretest	8.2 (4.9)		—		—
	Posttest	3.9 (4.2)		0.86 (0.49 to 1.39)		<.001
	3-month follow-up	3.7 (3.9)		0.91 (0.5 to 1.41)		<.001
	6-month follow-up	3.7 (4.6)		0.92 (0.49 to 1.44)		<.001
**CAQ attention**						
	Pretest	10.8 (2.5)		—		—
	Posttest	5.7 (2.4)		2.05 (1.33 to 2.97)		<.001
	3-month follow-up	5.8 (2.8)		2.03 (1.26 to 3.01)		<.001
	6-month follow-up	5.8 (3.3)		2.03 (1.15 to 3.09)		<.001
**MCS-12^g^**						
	Pretest	37.8 (9.8)		—		—
	Posttest	47.9 (9.5)		1.03 (0.6 to 1.54)		<.001
	3-month follow-up	47.0 (11)		0.94 (0.25 to 1.55)		<.001
	6-month follow-up	47.9 (10.8)		1.03 (0.39 to 1.62)		<.001
**PCS-12^h^**						
	Pretest	50.9 (8.3)		—		—
	Posttest	52.5 (6.4)		0.18 (–0.4 to 0.5)		.26
	3-month follow-up	50 (6.7)		0.11 (0.18 to 0.87)		.49
	6-month follow-up	51 (7.5)		0.01 (–0.51 to 0.34)		.95
**BSQ^i^**						
	Pretest	35.3 (8.9)		—		—
	Posttest	28.0 (8.7)		0.81 (0.34 to 1.46)		<.001
	3-month follow-up	26.0 (7)		1.04 (0.53 to 1.59)		<.001
	6-month follow-up	27.1 (8.5)		0.91 (0.44 to 1.63)		<.001
**PSS-4^j^**						
	Pretest	5.7 (3.2)		—		—
	Posttest	4.2 (2.9)		0.50 (0.26 to 0.89)		.01
	3-month follow-up	5.1 (3.5)		0.20 (–0.23 to 0.73)		.28
	6-month follow-up	4.4 ^m^ (2.9)		0.43 (0 to 0.95)		.02
**GLSTPAQ^k^**						
	Pretest	32.1 (24.1)		—		—
	Posttest	49.3 (24.5)		0.71 (0.34 to 1.18)		.01
	3-month follow-up	55.6 (42.2)		0.97 (0.49 to 2.28)		.001
	6-month follow-up	53.4 (33.3)		0.88 (0.45 to 1.83)		.002
**PHQ-9^l^**						
	Pretest	5.8 (2.8)		—		—
	Posttest	3 (2.6)		1.03 (0.58 to 1.52)		<.001
	3-month follow-up	3.8 (3.9)		0.71 (–0.27 to 1.32)		.02
	6-month follow-up	3.8 (3.9)		0.73 (–0.06 to 1.34)		.01
**GAD-7^m^**						
	Pretest	6.5 (3.2)		—		—
	Posttest	4.2 (3.9)		0.73 (0.17 to 1.42)		.008
	3-month follow-up	3.8 (3.4)		0.84 (0.24 to 1.47)		.003
	6-month follow-up	3.3 (3.7)		1.02 (0.11 to 1.59)		<.001

^a^Within-group effect size.

^b^95% CIs are based on 5000 bootstrap replications.

^c^AFEQT-PVC: Atrial Fibrillation Effects on Quality of Life adapted for PVCs.

^d^Not applicable; all assessments at all other time points were compared to the pretest level.

^e^SCL: Symptoms Checklist.

^f^CAQ: Cardiac Anxiety Questionnaire.

^g^MCS-12: 12-Item Short-Form Survey mental health subscale.

^h^PCS-12: 12-Item Short-Form Survey physical health subscale.

^i^BSQ: Bodily Symptoms Questionnaire.

^j^PSS-4: Perceived Stress Scale.

^k^GLSTPAQ: Godin Shepard Leisure Time Physical Activity Questionnaire; one outlier was removed from this analysis.

^l^PHQ-9: Patient Health Questionnaire 9-item scale.

^m^GAD-7: Generalized Anxiety Disorder 7-item scale.

### ECG Analyses

The ECG analyses (Table S1 in Multimedia Appendix 1) did not show any significant change in the objective PVC burden at post treatment (*P*=.87) or at 6-month follow-up (*P*=.21). However, we observed a statistically significant decrease in self-reported PVC symptoms (*P*=.006) as reported by tapping the patch-recorder symptom indicator when experiencing symptoms of PVCs at posttreatment assessment. These effects were sustained at 6-month follow-up (*P*=.003).

### Mediation Analysis

The potential mediators and outcomes were measured weekly for 9 consecutive weeks during treatment. We collected a mean number of 17.7 observations per week out of 19 possible observations, and no less than 16 for any week (week 5). Table S2 in Multimedia Appendix 1 shows the estimated indirect effects (ab-products) and their 95% CIs for the three mediators when tested separately and in competition in a multiple mediator model.

In the single mediator analysis, the following measures had statistically significant ab-products: CAQ fear (0.64), CAQ attention (0.87), CAQ avoidance (1.01), and PSS-4 (0.21). This indicated a mediating effect of both symptom preoccupation and stress sensitivity on the main outcome AFEQT-PVC. The effect of physical activity based on the GSLTPAQ (–0.01) was not statistically significant, regardless of whether or not the outlying participant was included in the analysis. When allowing the measures to compete in explaining the change in outcome in AFEQT-PVC in a multiple mediator analysis, the ab-product of PSS-4 (0.12) was statistically significant but substantially lower than that of the attention (0.42) and avoidance (0.80) subscales of the CAQ, which also remained statistically significant. The fear subscale of the CAQ (0.14) was nonsignificant in the multiple mediator analysis.

### Treatment Satisfaction

In total, 18 out of 19 participants (95%) reported that they were very satisfied (13/19, 68%) or satisfied (5/19, 26%) with the treatment. The mean score on the CSQ measure was 28.9 (SD 3.2) of a maximum 32 points, which indicated a high level of satisfaction with the treatment.

### Changes in Medication and Cardiac Health

At post treatment, 4 of the 19 (21%) participants reported changes in their cardiac medication. Three participants quit using beta-blockers and one participant reported an increase in the use of beta-blockers. At the 6-month follow-up, 6 participants (32%) reported changes in cardiac medication; 1 (5%) quit using beta-blockers, 1 (5%) decreased the use of beta-blockers, and 4 (21%) increased their use of beta-blockers (including one participant who started using beta-blockers to treat hypertension rather than symptoms of PVCs). At 6-month follow-up, 1 (5%) participant had undergone invasive therapy (catheter ablation) and 1 (5%) participant reported slightly increased blood pressure. In addition, 1 (5%) participant had episodes of paroxysmal AF based on the analyses of the ECG data after the 6-month follow-up.

### Adverse Events

At post treatment, among the 19 participants, 2 (11%) reported adverse events from engaging in the study. One of the participants (5%) reported increased stress and worry from participating in the study and rated the negative impact of this event as of mild severity (1 out of 3) at the time of the event as well as on the residual discomfort from the event. The other participant reported two adverse events during treatment consisting of elevated cardiac symptoms, rated as having the highest severity (3 out of 3) at the time of the events as well as on residual discomfort. No other adverse events were reported at the 3-month follow-up. At the 6-month follow-up, 1 (5%) participant reported an adverse event consisting of an episode of increased frequency of palpitations, rated as of medium severity at the time of the event (2 out of 3) and of mild severity on residual discomfort from the event (1 out of 3).

## Discussion

### Principal Findings

To our knowledge, this is the first study to evaluate the potential efficacy and feasibility of PVC-CBT for symptom preoccupation in patients with symptomatic idiopathic PVCs. Substantial improvements were found on the primary outcome measure AFEQT-PVC with respect to PVC-specific QoL and self-reported PVC symptoms. We also observed large reductions in cardiac-related fear and hypervigilance and avoidance behavior as measured by the CAQ. Furthermore, medium to large improvements were observed on all other secondary measures, except for the physical health domain of the SF-12, which remained unchanged. ECG analyses showed a significant reduction in self-reported PVC symptoms, although the objective PVC burden was unchanged. All posttreatment results were sustained at 3- and 6-month follow-ups, with high adherence and participant satisfaction and few adverse events reported. These results are comparable to the results for exposure-based AF-CBT delivered face to face [11] and via the internet [12,14] as well as for CBT for functional somatic disorders [6,7,33-35]. The exploratory mediation analyses indicated that symptom preoccupation had a mediating effect on the impact of PVC-CBT on PVC-specific QoL and self-reported PVC symptoms. These results are consistent with previous results of AF-CBT [12,14] and of CBT for other somatic conditions [36-38]. Sustained improvements in PVC-specific QoL (AFEQT-PVC) and self-reported PVC symptoms (SCL and tapping the ECG) were observed despite the lack of change in the objective PVC burden, providing support for further investigation on the proposed role of symptom preoccupation as a potential maintaining mechanism of symptom focus and impairment in PVCs.

The promising findings of this pilot study, with large improvements in the outcome measures, high treatment adherence, and reported satisfaction with treatment as well as the limited report of adverse events, encourage further research into the efficacy of CBT in patients with symptomatic PVCs. The use of videoconferencing and text-based material delivered online in this study enabled access to treatment for patients who do not otherwise benefit from current treatment regimens and for patients in rural areas. If the results of this study can be replicated in further studies, PVC-specific CBT may be made available to larger groups of patients, irrespective of their geographical location. In the future, as digital devices are increasingly used in the detection and follow-up of patients with arrhythmias [39], online CBT may be combined with other telemedicine applications as part of a remote management strategy in patients with PVC or other arrhythmic conditions.

### Limitations

There are several limitations to this study, which should be considered when interpreting the results. The within-group design with a lack of control group precludes deriving a firm conclusion as to whether the results are true effects of the intervention or caused by extraneous variables such as the passage of time, expectancy of improvement, or attention from a caregiver. Another limitation is the reliance on self-reported measures, which could raise validity concerns due to the subjective nature of self-reporting. Nevertheless, the use of self-reported outcomes of QoL in arrythmia intervention research is endorsed [40]. All self-reported measures used in this study are well-validated, except for the main outcome measure (AFEQT-PVC), which has not been validated for the PVC population. Unfortunately, to our knowledge, there is no validated PVC-specific QoL questionnaire, and while the AFEQT is a well-validated measure for AF-specific QoL and patients with PVCs show a similar symptom presentation and arrhythmia-related disability to those of patients with AF, important aspects of QoL among patients with PVCs may not be adequately reflected by this measure. In addition, the ECG patch measurement period of 5 days may have been too short to measure the objective PVC burden accurately. A more reliable measure could have been obtained by using an implantable loop recorder. However, this was considered too intrusive for a secondary outcome measure. Inferences on generalizability are also limited by the small sample size and the skewed sex distribution with a majority of female participants (14/19, 74%). A possible selection bias may have been introduced due to recruitment mainly from advertisements on social media, which may affect the generalizability to patients in routine care. However, the treatment is designed for patients with symptom preoccupation who are willing to engage in psychological intervention and thus the selected recruitment approach may also have lowered the threshold for seeking treatment. A majority (13/19, 68%) of the participants had previous experience of psychological treatment, which may have made them more susceptible to the CBT treatment.

### Conclusions

The main objective of this pilot study was to investigate the potential efficacy of PVC-CBT for patients with symptomatic idiopathic PVCs delivered by videoconference together with online text-based modules and homework assignments. The large improvements on PVC-specific QoL and the indication that the effect on PVC-specific QoL was mediated by reductions in symptom preoccupation suggest potential efficacy of exposure-based CBT targeting symptom preoccupation for this patient group. Randomized controlled trials are warranted to confirm our findings in larger patient samples.

## Appendix

Multimedia Appendix 1 Detailed results of the electrocardiogram (ECG; Table S1) and exploratory mediation (Table S2) analyses.

## Abbreviations

AF: atrial fibrillation
AF-CBT: atrial fibrillation–specific cognitive behavioral therapy
AFEQT: Atrial Fibrillation Effects on Quality of Life
AFEQT-PVC: Atrial Fibrillation Effects on Quality of Life adapted to premature ventricular contraction
BSQ: Body Sensations Questionnaire
CAQ: Cardiac Anxiety Questionnaire
CBT: cognitive behavioral therapy
CSQ: Client Satisfaction Questionnaire
ECG: electrocardiogram
GAD-7: Generalized Anxiety Disorder scale
GSLTPAQ: Godin-Shepard Leisure Time Physical Activity Questionnaire
MCS-12: mental health–related quality of life subscale of the Short-Form Health Survey
PCS-12: physical health–related quality of life subscale of the Short-Form Health Survey
PHQ-9: 9-item Patient Health Questionnaire
PSS-4: Perceived Stress Scale
PVC: premature ventricular contraction
PVC-CBT: premature ventricular contraction–specific cognitive behavioral therapy
QoL: quality of life
SCL: Symptoms Checklist
SF-12: Short-Form Health Survey
TREND: Transparent Reporting of Evaluations with Nonrandomized Designs

## Data Availability

The data sets generated during and/or analyzed during this study are not publicly available due to European Union law regulating sensitive personal data. Access to data requires approval from the Swedish Ethical Review Authority and data sharing agreement with Karolinska University Hospital.
