# Supplementary material for: Cognitive Behavioral Therapy for Symptom Preoccupation Among Patients With Premature Ventricular Contractions: Nonrandomized Pretest-Posttest Study
Source: JMIR Cardio. 2024 May 7;8:e53815. doi: 10.2196/53815 (PMC11109856; doi:10.2196/53815)
Supplement: Multimedia Appendix 1 [file cardio_v8i1e53815_app1.docx]

Table S1. ECG measurements: Changes from pre-treatment assessment in objective PVC burden (mean PVC/24h) as measured with continuous three- channel ambulatory ECG and changes from pre-treatment assessment in self-reported PVC burden (mean tapping/24h) as reported by tapping on the patch recorder when experiencing PVCs.

| **Variables** | **Estimated^a^ Mean PVC/24h^a^** | **[95% Conf. Interval]** | **IRR^b^** | **Robust Std.err** | **P>\|z\|** | **[95% Conf. Interval]** |
| --- | --- | --- | --- | --- | --- | --- |
| **Objective PVC burden** |  |  |  |  |  |  |
| Pre-treatment | 2583 | (1047, 6370) |  |  |  |  |
| Post-treatment | 2479 | (554, 4405) | 0.96 | 0.24 | .87 | (0.59, 1.56) |
| 6-month follow-up | 1667 | (97, 3236)) | 0.65 | 0.23 | .21 | (0.33, 1.28) |
| **Subjective PVC burden** |  |  |  |  |  |  |
| Pre-treatment | 19 | (1,37) |  |  |  |  |
| Post-treatment | 8 | (3,13) | 0.41 | 0.13 | .006 | (0.22, 0.78) |
| 6-month follow-up | 5 | (1,10) | 0.28 | 0.12 | .003 | (0.13, 0.64) |

^a^Estimated mean PVCs per 24 hours and corresponding 95% confidence intervals are reported for objective PVCs, and self-reported PVCs at pre-treatment, post-treatment, and six-month follow-up.

^b^IRR with 95% confidence intervals, and associated p-values were calculated with a Poisson GEE model. IRR is the estimated change from pre-treatment assessment and reported as significant when the 95% confidence intervals are not containing zero.

Table S2. Indirect effects, *ab*-product, of the five tested mediators of the effect of treatment week on the primary outcome measure AFEQT-PVC.

|  | Single mediator  analysis | | Multiple mediator  analysis | |
| --- | --- | --- | --- | --- |
| Mediator | *ab* | Bootstrapped 95%CI | *ab* | Bootstrapped 95% CI |
| CAQ fear | 0.64^a^ | (0.30 to 1.04) | 0.14 | (-0.19 to 0.43) |
| CAQ avoidance | 1.0 ^a^ | (0.56 to 1.83) | 0.80^a^ | (0.44 to 1.47) |
| CAQ attention | 0.87^a^ | (0.49 to 1.42) | 0.42^a^ | (0.07 to 0.79) |
| GSLTPAQ^b^ | -0.01 | (-0.08 to 0.01) | -0.03 | (-0.16 to 0.03) |
| PSS4 | 0.21^a^ | (0.10 to 0.41) | 0.12 | (0.04 to 0.28) |

^a^Statistically significant indirect effects, *ab*-products, based on their respective 95% confidence intervals not containing zero. Abbreviations: AFEQT-PVC = Atrial Fibrillation Effect on Quality-of-Life – Adapted for PVCs, CAQ = Cardiac Anxiety Questionnaire, GSLTPAQ = Godin Shepard Leisure Time Physical Activity Questionnaire, PSS-4 = Perceived stress scale - 4 item version.

^b^Analysis including the outlier. Analysis with the outlier removed did not change the result.
